# Oligonucleotide Selective Detection by Levitated Optomechanics

**DOI:** 10.1021/acsnanoscienceau.5c00128

**Published:** 2025-10-20

**Authors:** Timothy Wilson, Owen J. L. Rackham, Hendrik Ulbricht

**Affiliations:** † School of Ocean and Earth Science, 514186 University of Southampton, Waterfront Campus, European Way, Southampton SO14 3ZH, United Kingdom; ‡ School of Biological Sciences, 7423 University of Southampton, Southampton SO17 1BJ, United Kingdom; ¶ School of Physics and Astronomy, University of Southampton, Southampton SO17 1BJ, United Kingdom

**Keywords:** Levitated Optomechanics, Optical Trapping, Silica Nanoparticles, Oligonucleotide Detection, Biosensing

## Abstract

This study examines the detection of oligonucleotide-specific signals in sensitive optomechanical experiments. Silica nanoparticles were functionalized using ZnCl_2_ and 25-mers of single-stranded deoxyadenosine and deoxythymidine monophosphate which were optically trapped by a 1550 nm wavelength laser in vacuum. In the optical trap, silica nanoparticles behave as harmonic oscillators, and their oscillation frequency and amplitude can be precisely detected by optical interferometry. The data was compared across particle types, revealing differences in frequency, width, and amplitude of peaks with respect to motion of the silica nanoparticles which can be explained by a theoretical model. Data obtained from this platform was analyzed by fitting Lorentzian curves to the spectra. Dimensionality reduction detected differences between the functionalized and nonfunctionalized silica nanoparticles. Random forest modeling provided further evidence that the fitted data were different between the groups. Transmission electron microscopy was carried out but did not reveal any visual differences between the particle types.

## Graphical abstract

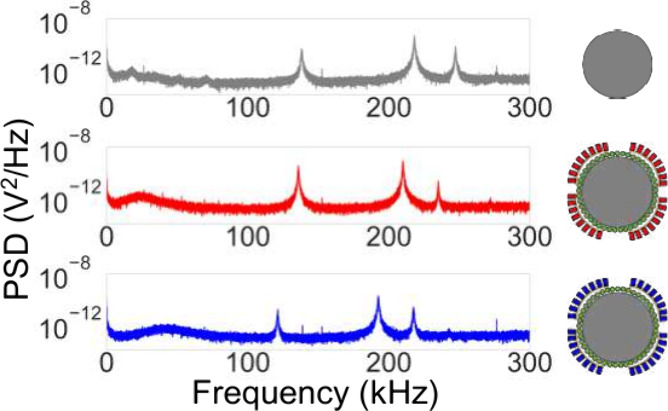

Detecting and differentiating DNA strands has applications in the fields of medicine, data storage and evolutionary biology.^1–3^ It is therefore of interest to develop methods to study DNA with greater speed and accuracy. The Sanger sequencing method was published in 1977 ^4^ with parallelization and high-throughput now standard in modern techniques.^5^ This research presents an alternative method based upon the optical properties of DNA nucleotides.

Optical trapping was originally observed by Arthur Ashkin in 1970 to contain micron-sized particles.^6^ This technique, known as optical tweezing, has since found applications in biosensing and live cell imaging in a solution.^7,8^ Optical trapping in vacuum is commonly referred to as levitated optomechanics^9^ and is the approach used in this work. By levitated optomechanics, it is possible to measure tiny forces of trapped particles on the order of 10^–20^ N,^10^ leading to the notion that a trapped particle functionalized with DNA might be distinguished from those particles that do not have surface modifications.

Silica is routinely used in optical trapping due to its greater refraction than water at near-infrared wavelengths, a property required for stable optical trapping in a water medium.^11^ Silica nanoparticles were used early in the development of optical trapping by Ashkin and Dziedzic where they demonstrated levitation of 20 μm diameter silica nanoparticles at a pressure of 1 mbar.^12–14^ Factors important to consider in the choice of material include high polarizability and low absorption at the wavelength of the source. Silica meets these criteria at the 1550 nm wavelength.^15^ The properties of silica nanoparticles, and their ability to be functionalized with DNA, are the reasons why they were used in this study.

The process of DNA adsorption onto the surface of nanoparticles remains rarely studied.^16^ Metal ions are vital in living organisms, the regulation of biological processes^17^ and as cofactors of DNAzymes.^18^ One paper examined methods to functionalize silica nanoparticles with short fluorescein-tagged DNA strands using different metal ions as binding agents.^19^ They found that Zn^2+^ ions from a ZnCl_2_ solution at a concentration of 1 mM was one of the best adsorption metal ions to bind fluorescein tagged 25-mer deoxyadenosine monophosphate oligonucleotides to silica nanoparticles. This study did not carry out optical trapping of silica nanoparticles, however it did provide a foundation for the method of functionalization used in this research.

The research presented here uses the method of particle preparation for release into the optical trap in a vacuum as shown in Figure 1. The sonicator is used to reduce particle aggregation when released into the optical trap from the nebulizer.

**Figure 1:**
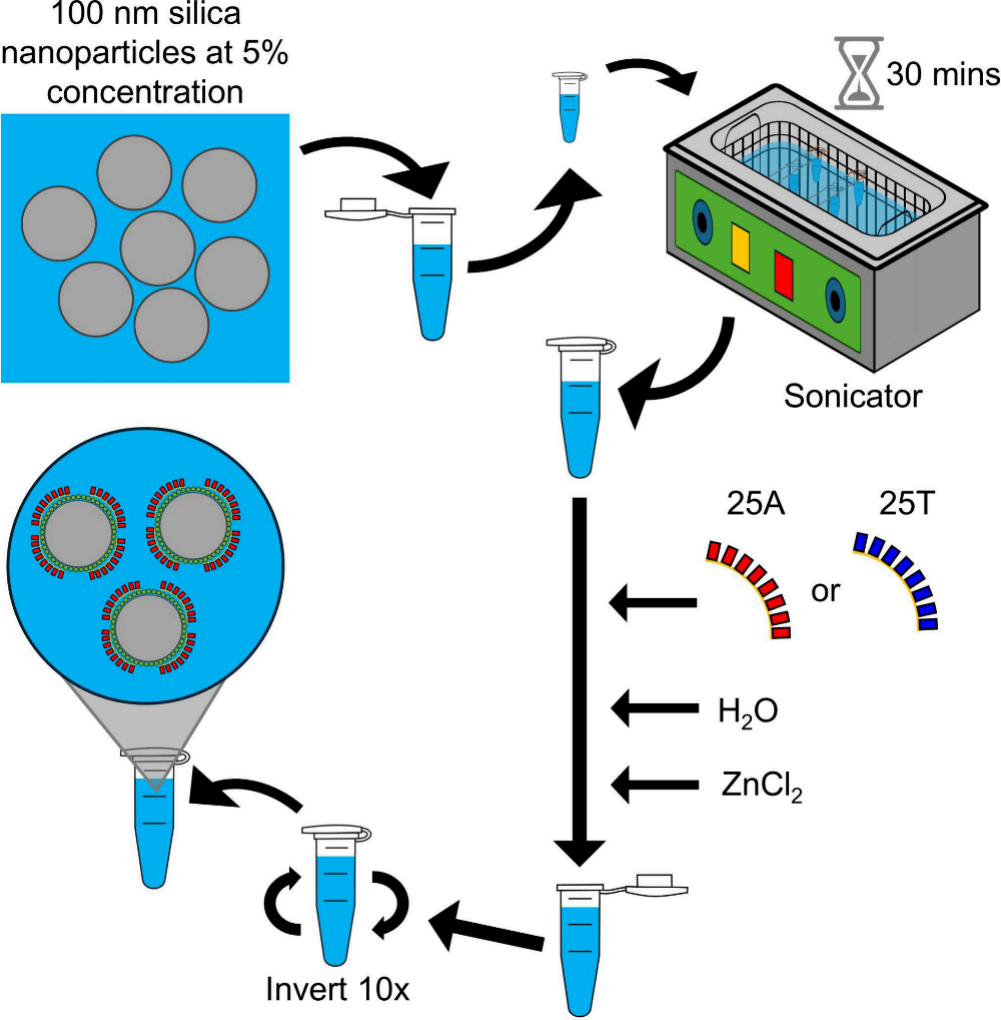
Functionalization: the process of functionalizing silica nanoparticles with 25-mer deoxyadenosine monophosphate (25A) or 25-mer deoxythymidine monophosphate (25T) oligonucleotides, water, and ZnCl_2_ solution. Eppendorf tube images are provided by Labicons.

Here we present the technique of optical trapping under vacuum to explore the detection of oligonucleotide functionalization differences between groups of silica nanoparticles. Silica nanoparticles with no surface modifications (standard silica nanoparticles) were tested as a reference, then 25-mer deoxyadenosine monophosphate (25A) and 25-mer deoxythymidine monophosphate (25T) oligonucleotides were added with ZnCl_2_ as a binding agent to the silica nanoparticles with concentrations of ZnCl_2_ changed for the 25T variant. The raw data for each particle type was compared. Uniform Manifold Approximation and Projection (UMAP) dimensionality reduction and random forest analysis were used to examine how well these groups of particles could be classified. Transmission Electron Microscopy (TEM) imaging was also used to attempt to visualize differences. Collectively, this provides evidence for the selective detection of these silica nanoparticles using levitated optomechanics.

The nanoparticles were released into a vacuum chamber, and when trapped, the chamber was pumped down to a consistent 3.5 mbar within the range of error of the pressure gauge. The Power Spectral Density (PSD) waveforms were recorded on an oscilloscope. After the data were collected, the particles were filtered to remove outliers. The process of calculating numerical columns and outlier removal was carried out by fitting Lorentzian curves to the *f*_1_, *f*_2_ and *f*_3_ frequency peaks, which correspond to the *z*, *x* and *y* degrees of motion, respectively, using the tool, Optoanalysis.^20^ The PSD, *S*_*xx*_(ω), as seen in Figure 2 has units of V^2^/Hz and can be written as

1 $$S_{xx}\left(\omega \right)=\gamma^{2}\frac{k_{B}T_{0}}{\pi m}\frac{\Gamma_{0}}{{\left(\omega_{0}^{2}-\omega^{2}\right)}^{2}+\omega^{2}\Gamma_{0}^{2}}$$

where γ is the conversion factor, *k*_B_ is the Boltzmann constant, *T*_0_ is the temperature of the environment, *m* is mass of the particle, Γ_0_ is the damping rate, ω_0_ is the natural angular frequency, and ω is the angular frequency at which the PSD is calculated. Equation 1 can be simplified to the experimental data, *S*_*xx*_^exp^, as below:

2 $$S_{xx}^{\exp}=\frac{A}{(B^{2}-\omega^{2}{)}^{2}+\omega^{2}C^{2}}$$

where $A=\frac{\gamma^{2}k_{B}T_{0}\Gamma_{0}}{\pi m}$ , *B* = ω_0_ and *C* = Γ_0_ are free fit parameters. The conversion factor γ converts the PSD to units of m^2^/Hz and can be calculated as follows in eq 3:

3 $$\gamma =\sqrt{\frac{A}{C}\frac{\pi m}{k_{B}T_{0}}}$$

**Figure 2:**
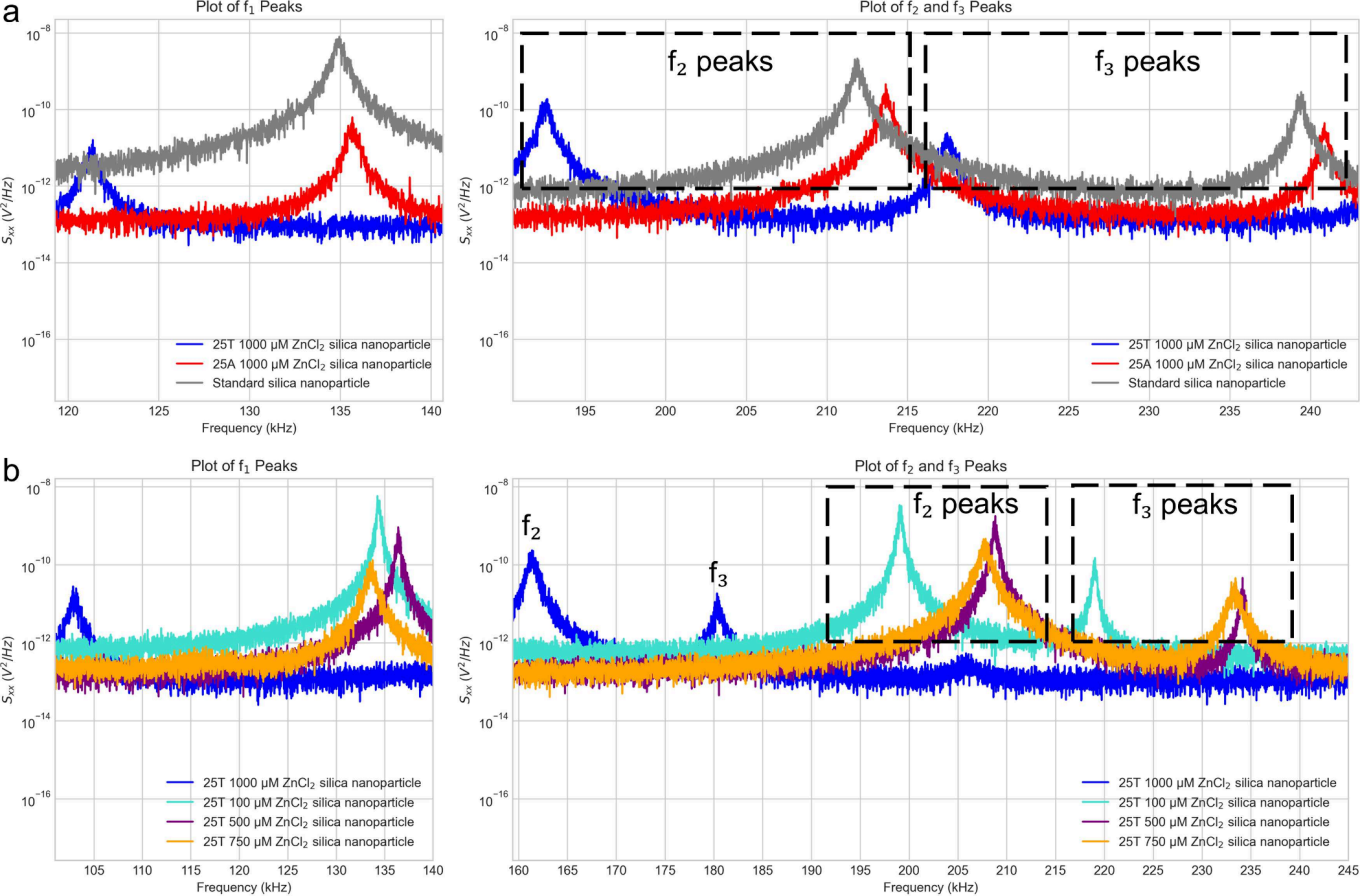
PSD plots of groups of selected particle types with frequency peaks *f*_1_, *f*_2_ and *f*_3_ displayed. (a) Plots of the *f*_1_, *f*_2_ and *f*_3_ peaks for one particle of each type: 25A silica nanoparticle at a 1000 μM ZnCl_2_ concentration (red) and 25T silica nanoparticle at a 1000 μM ZnCl_2_ concentration (blue) and for a standard silica nanoparticle (gray). (b) Plots of the *f*_1_, *f*_2_ and *f*_3_ peaks for one particle of each type: 25T silica nanoparticle at a 1000 μM ZnCl_2_ concentration (blue), 25T silica nanoparticle at a 100 μM ZnCl_2_ concentration (turquoise), 25T silica nanoparticle at a 500 μM ZnCl_2_ concentration (purple), and 25T silica nanoparticle at a 750 μM ZnCl_2_ concentration (orange).

The nanoparticle is assumed to be in a thermal equilibrium where *T*_0_ = 300 K. The radius is derived from the fit parameter of the Lorentzian curve from the PSD in eq 4 as in the literature:^15^

4 $$r=0.619\frac{9\pi }{\sqrt{2}}\frac{\eta_{air}d^{2}}{\rho k_{B}T_{0}}\frac{P_{gas}}{C}$$

where *r* is the particle radius, η_air_ is the viscosity of air, *d* is the diameter of the atmospheric particles, *P*_gas_ is the pressure measured from the pressure sensor and ρ is the particle material density. Assuming the particle to be spherical, the mass of the particle can be calculated using $m=\frac{4}{3}\pi r^{3}$ .

The resulting parameters were filtered and outliers removed. An outlier is defined as being greater than 1.5 interquartile ranges from the median for any numerical column. The complete raw data set is available in the . There are considerable variances in the size of silica nanoparticles, which is the primary reason for this filtering. The data analysis presented here are with outliers removed.

The raw PSD data were compared for each silica nanoparticle type. One candidate was selected from each group, and the *f*_1_, *f*_2_ and *f*_3_ frequency peaks were displayed in sections for comparison. Both panels in Figure 2 highlight differences observed between the particles. In Figure 2a, there is distinct separation in the PSD regarding width, amplitude and frequency of all peaks. Figure 2b shows differences between most frequency peaks for the 25T functionalized silica nanoparticles at different ZnCl_2_ concentrations, the exception being the similarity between the 25T silica nanoparticles at 500 μM and 750 μM ZnCl_2_ concentrations. These PSDs are very similar in frequency, amplitude and width at the *f*_2_ and *f*_3_ peaks.

A physics explanation for the observed trap frequency shift for the oligonucleotide base-coated silica nanoparticle in the levitated optomechanical trap is described in the . This model, which ignores the ZnCl_2_ salt layer surrounding the surface of the silica nanoparticles, finds that the polarizability-to-mass ratio (α/*m*) changes depending on the oligonucleotide. It is this change in α/*m*, not mass, which is responsible for the frequency shift. The measure of α/*m* is routinely used in metrology to complement mass spectrometry and sort fullerenes and polypeptides.^21,22^ The estimates show that there is about a 1 kHz frequency shift per monolayer of DNA base molecule. There is a difference between adenine and thymine, and it is estimated that there is a frequency shift of 30 mHz corresponding to a single adenine molecule when compared to a single thymine base.

Dimensionality reduction is often used in the field of machine learning to compress a data set with many features, or columns, into a manageable number of components.^23^ This technique decreases the complexity of these data and improves the accuracy of classification.^24^ UMAP is a dimensionality reduction technique that can be run in a supervised learning mode to maximize the space between known classes in low-dimensional space that have features which are nonlinearly correlated.^25,26^ This method was selected for its flexibility in the analysis of any type of high-dimensional data.^27^ Applying UMAP to the data collected allows examination of the differences between groups in a two-dimensional graphical representation.

The random forest technique is an ensemble machine learning algorithm that is effective as a generalized classification and regression model.^28^ Ensemble techniques have a greater accuracy than other machine learning methods, such as Support Vector Machines and K-Nearest Neighbors, because a group of classifiers tends to perform more accurately than an individual.^29,30^ A random forest is a group of decision trees, where each tree provides its own classification, and these are considered collectively through a vote to reach a classification consensus. The overall random forest algorithm considered the classification with the greatest number of votes from all the trees in the forest.^31^ Usefully, the random forest can return a measure of feature importance.^32^ Furthermore, the random forest model also produces an accuracy score, giving an indication of its performance. This is calculated using the Out of Bag Error which assesses the mean misclassification ratio of samples not used for training.^33^

To assess the performance of the random forest classifier in this experiment, a cross-validation technique was used. Accuracy scores from a single random forest run, particularly on a data set of small sample size, are challenging to interpret and often do not give a complete picture. Monte Carlo cross-validation (MCCV) is a method which is suitable for small sample sizes. It functions by randomizing the samples in each training and test data set and can be run for as many iterations as desired for robustness.^34^ MCCV can be applied to a random forest classification problem to validate the accuracy of the model, or optimize the features for use in future data.^35^ A recent study compared resampling methods and found that no technique was consistently better than the others.^36^

The PSD comparison of one particle from each class in Figure 2 suggests that there is a difference in optical properties between the silica nanoparticle groups. UMAP dimensionality reduction was used to plot the features of the particles from the Optoanalysis^20^ fitting in two dimensions. The key objective of this analysis was to determine if it is possible to detect a difference between standard and oligonucleotide-functionalized silica nanoparticle. The clustering depicted in Figure 3a for 25A and 25T silica nanoparticles at the same 1000 μM concentration of ZnCl_2_ suggests there is variation in the data that can be explained by silica nanoparticle status. The dimensionality reduction has considered all available features generated by the Lorentzian curve fitting to the raw data. There is a separation between the three particle types, with no overlap between these groups. Next, the UMAP method was applied to investigate if changing the concentration of ZnCl_2_ has a detectable effect on 25T functionalization to the surface of silica nanoparticles. The separation observed in Figure 3b between all concentrations of ZnCl_2_ shows agreement with data from the literature^19^ in that different concentrations of the binding agent can cause measurable changes in the quantity of DNA on the surface of silica nanoparticles. Both 2-dimensional UMAP clustering figures demonstrate the effectiveness of this method in clustering individual groups and separating the particle type classes.

**Figure 3:**
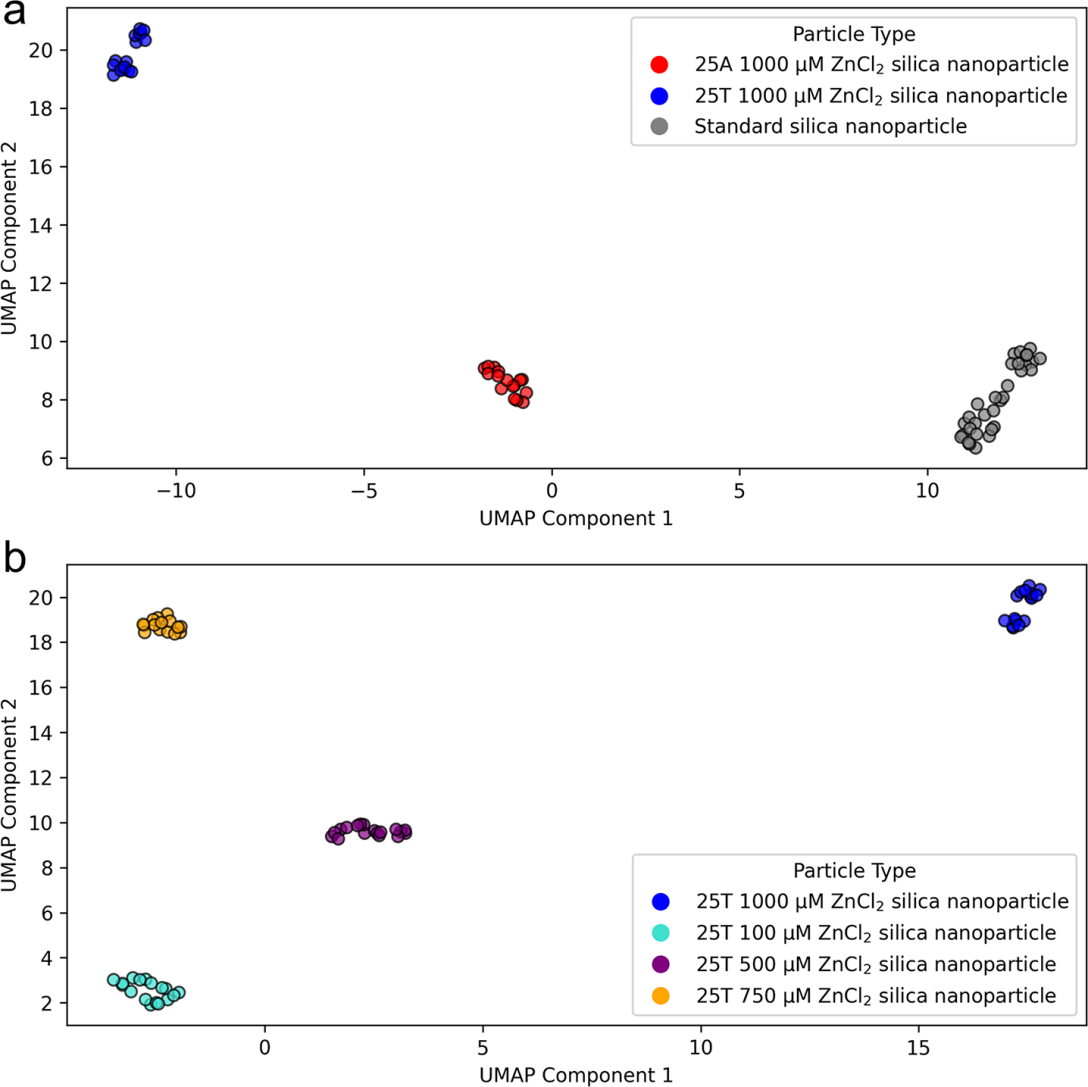
2-Dimensional UMAP analysis of optically trapped silica nanoparticles. (a) This UMAP scatter plot depicts the differences between the trapped 25A silica nanoparticles and 25T silica nanoparticles plotted alongside the standard silica nanoparticles. Both 25A silica nanoparticles and 25T silica nanoparticles use a 1000 μM concentration of ZnCl_2_. (b) This UMAP scatter plot demonstrates the similarities and differences between varying the concentrations of ZnCl_2_ on the binding of 25T to the silica nanoparticle surface. Both (a) and (b) use the following parameters of number of nearest neighbors = 50 and minimum distance = 0.0 to observe the global difference between particle types.

A random forest model was trained on the Optoanalysis tool parameters with outliers removed. The data sets were split into 80% for training and 20% for testing. Training parameters and features were iterated through, and the optimal combination was selected to the deliver the best model accuracy. Due to the limitation in size of the data sets, where the 25A and 25T silica nanoparticles at 1000 μM ZnCl_2_ and standard silica nanoparticles data has 64 entries, and the 25T silica nanoparticles at different ZnCl_2_ concentrations data have 66 entries, the MCCV method was utilized to give a more complete understanding of random forest performance. Figure 4a shows that the random forest model performs well over the 300 iterations, at best there is perfect accuracy, at worst it is 62% accurate, the mean accuracy is 87%. This shows agreement with the UMAP plot in Figure 3a with distinct clustering between particle types suggesting that classification not random. In Figure 4b the model has a good mean accuracy at 73%, with a minimum of 50% and maximum of 93%. An explanation for the weaker mean accuracy in Figure 4b compared to Figure 4a could be the similarity between the DNA binding for 25T 500 μM and 750 μM ZnCl_2_ silica nanoparticles.^19^ The ranking of mean feature importance in Figure 4c is in the same order for both models; however, the magnitude of importance varies. The *f*_2_ and *f*_3_ A parameters are most important, with the *f*_1_ and *f*_2_ radii coming in last. This suggests a consistent importance of these features to classify the particle groups. The *f*_3_ radius was not used in any iteration and is therefore not shown.

**Figure 4:**
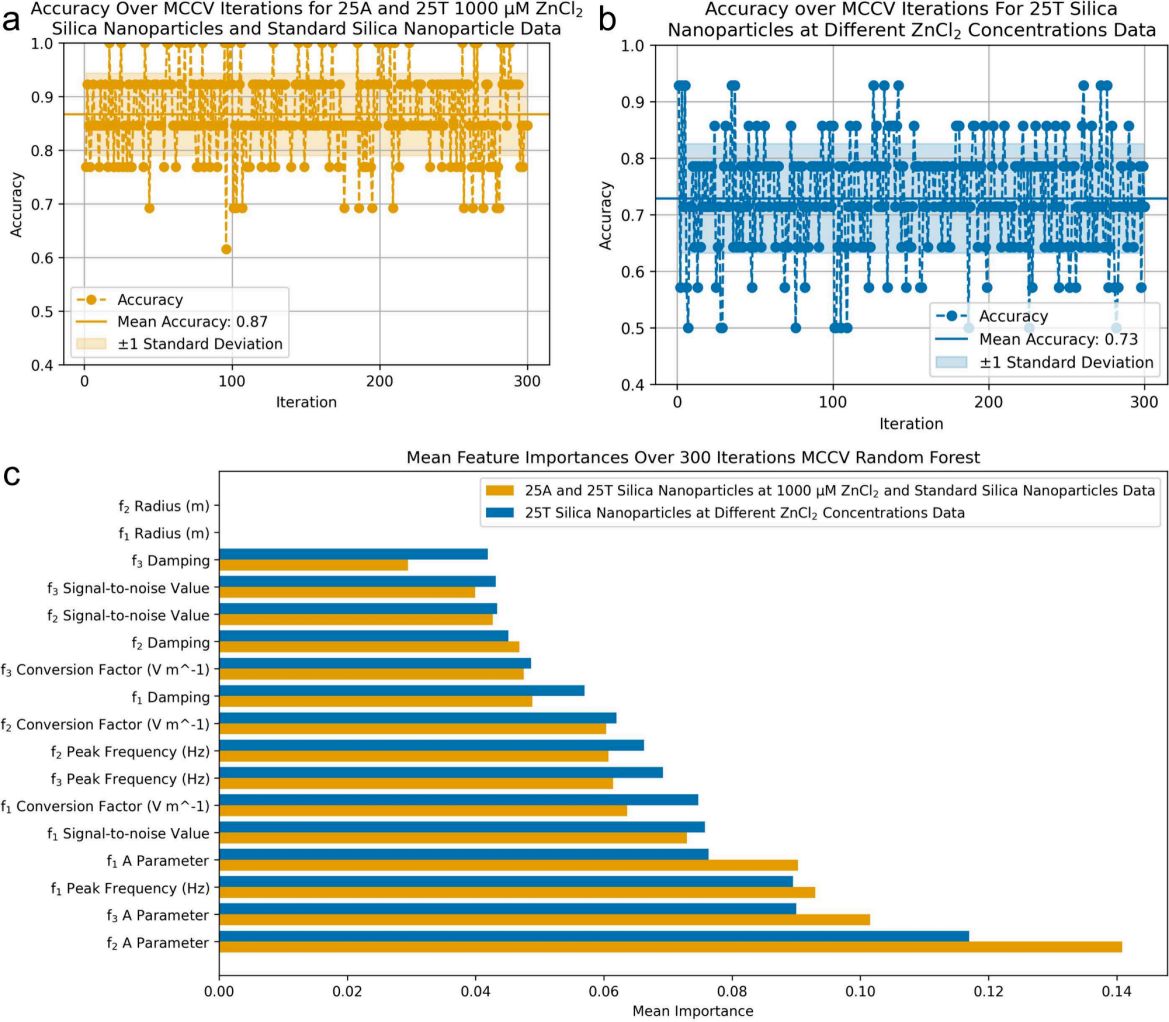
Random forest results comparing two groups of silica nanoparticles. (a) Random forest model accuracy for the 25A and 25T silica nanoparticles at 1000 μM ZnCl_2_ and standard silica nanoparticle data over 300 MCCV iterations. (b) Random forest model accuracy for the 25T silica nanoparticles at different ZnCl_2_ concentration data over 300 MCCV iterations. (c) Mean feature importances of both data sets over 300 MCCV iterations.

Transmission electron microscopy (TEM) visualization of DNA molecules is challenging.^37^ One study was able visualize DNA duplex features such as the major groove, minor groove and helix pitch using high-resolution TEM at 70 keV; however, further information about base sequence was challenging to infer.^38^ There are heavy metal staining methods to visualize DNA with TEM including uranyl acetate, but this is difficult due to its radioactivity.^39^ TEM imaging of silica nanoparticles is straightforward as the particles provide good contrast for visualization.^40^

All particle types that were tested in the optical trap were also imaged using TEM. The same sample preparation method was used as shown in Figure 1. The images presented in Figure 5 show the variance in particle size and shape. This is the reason why outliers were removed in the data analysis since considerable differences could be due to physical rather than optical properties. Despite sonicating the particle solutions, there are still clusters of particles present during imaging. The speckled white surface texture is representative of the surface roughness of the particles; they are not perfect spheres. There were no clear visual differences between the nanoparticle groups.

**Figure 5:**
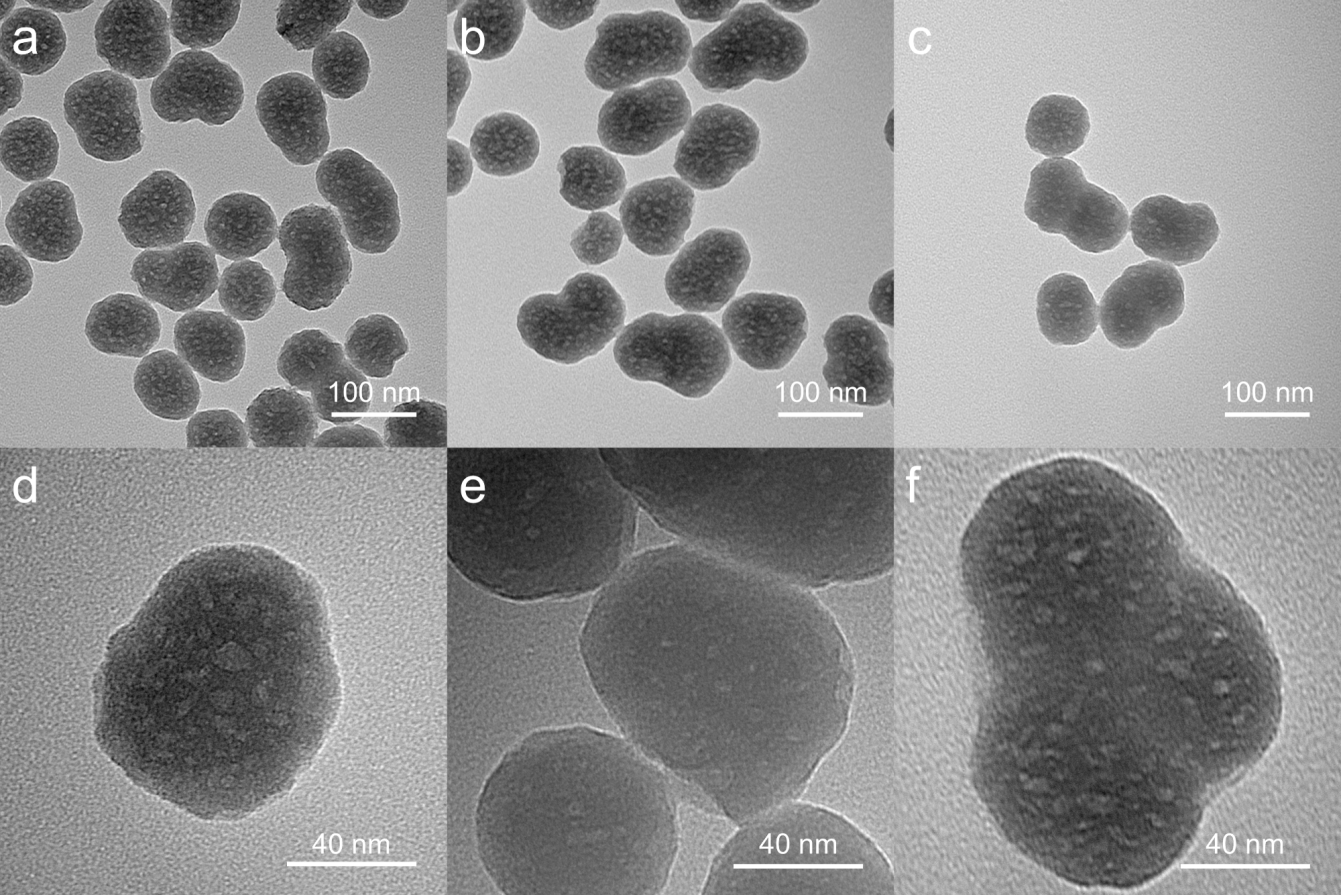
TEM images of silica nanoparticles. (a) 25T 100 μM ZnCl_2_ silica nanoparticles. (b) 25T 500 μM ZnCl_2_ silica nanoparticles. (c) 25T 1000 μM ZnCl_2_ silica nanoparticles. (d) Standard silica nanoparticle. (e) 25A 1000 μM ZnCl_2_ silica nanoparticles. (f) 25T 750 μM ZnCl_2_ silica nanoparticle. a–c are at 100,000× magnification. d–f are at 600,000× magnification.

The analysis performed in this study found detectable differences in optical properties between the types of silica nanoparticles. Taking one example from each particle group and plotting the raw data demonstrates that the Lorentzian curve fitting to produce the UMAP plots and random forest models is based upon foundational differences in their inputs. The UMAP result indicates that there are differences in the features between the silica nanoparticle classes that cause observable separation. The random forest modeling and MCCV iterations reveal that these classes can be identified with high mean accuracies of 0.87 and 0.73. Finally, the TEM imaging suggests that there is not an observable difference between the groups of silica nanoparticles.

To overcome the indistinguishable differences between silica nanoparticle groups using TEM, future work could include the use of Scanning Electron Microscopy and Energy Dispersive X-ray Spectroscopy (SEM-EDS). This technique can detect the presence of elements in a sample of atomic number greater than 11,^41^ so would detect the addition of zinc and phosphorus on the DNA-functionalized silica nanoparticles when compared to standard silica nanoparticles.

There is a limitation in data quantity as can be seen in the UMAP plots in Figure 3. To extend this analysis, a larger data set would need to be generated. However, the MCCV result regarding iterating through different variations of training and testing data does give confidence that there is a real difference in the data that are classified by the random forest model.

Having a classifier to distinguish between DNA strands could have applications in diagnostic testing, particularly where current sequencing technologies are challenged by GC-rich or repetitive regions.^42,43^ 25A and 25T oligonucleotides were selected in this study because they have been shown to bind well with Zn^2+^ to silica nanoparticles,^19^ however, they are also as different from each other as possible. Changing the bases to 24T with one A, swapping around the position of the A nucleotide in the strand, or changing the strand length and sequence entirely would be necessary to demonstrate this approach for further applications. Another question not investigated in this study was the effect of duplex DNA. Experimenting with a 25A–25T duplex would also indicate if there was a uniqueness to the DNA structure. Perhaps the additional binding offered by another phosphodiester backbone or the greater mass would affect how the data is clustered.

In summary, this study could lead down several directions. The frequency shift of 30 mHz in the proposed model can be feasibly detected in future experiments and will need to be investigated. If this is proved experimentally, then the ability to detect individual mass changes due to differences in base sequence could have uses in determining mutational or methylation modifications to DNA strands.^44,45^ Further development requires the correct foresight into how this technology could be applied. Work and thought are needed to explore why these results are present. Exploration into the underlying cause of different optical properties may shed light on the reasoning behind the UMAP clustering and random forest classification. Bioinformatics is becoming an ever more essential component of modern medicine; perhaps this optical trap classifier will be another method in the toolkit.

## Supplementary Material





## Data Availability

Raw data used for this paper: https://doi.org/10.5258/SOTON/D3576.

## Notes

Materials and methods, estimates of the frequency shift for oligonucleotide adsorption onto silica in levitated optomechanics, and supporting references (PDF)
(ZIP)
