# Supplementary material for: Oligonucleotide Selective Detection by Levitated Optomechanics
Source: ACS Nanosci Au. 2025 Oct 20;6(1):28–34. doi: 10.1021/acsnanoscienceau.5c00128 (PMC12921583; doi:10.1021/acsnanoscienceau.5c00128)
Supplement: Supplementary file 1 [file ng5c00128_si_001.pdf]

Supporting Information for paper: Oligonucleotide Selective Detection by Levitated Optomechanics

Timothy Wilson[1], Owen J.L. Rackham[1], Hendrik Ulbricht[1*]

[1]University of Southampton, University Road, Southampton, SO17 1BJ, UK

*Correspondence: h.ulbricht@soton.ac.uk

Contents

1 Materials and Methods **2**
- 1.1 Particle preparation 2
- 1.2 Optical trap laser setup 3
- 1.3 Data collection and analysis 4
- 1.4 Process of TEM imaging 6

2 Estimates of the Frequency Shift for Oligonucleotide Adsorption onto Silica in Levitated Optomechanics **7**
- 2.1 Trap frequencies in optical dipole trap of polarizable particle 7
  - 2.1.1 Trap frequency formula for z-direction 7
  - 2.1.2 Computation and experiments for dielectric properties of oligonucleotides 7
- 2.2 Trap frequency change for oligonucleotide coated silica nanoparticles 8
- 2.3 Frequency shift for a single adenine/thymine molecule 8

Supplementary References **9**

1 Materials and Methods

1.1 Particle preparation

Deionized water was used in all experiments for dilution to ensure that no potential contaminants such as DNases would break down the oligonucleotides. All particle solutions were prepared using 100 μL of 100 nm diameter Corpuscular Silicon Dioxide Nanospheres at 5.0% concentration diluted to a final volume of 1100 μL. Following the addition of silica nanoparticles, Amcor M Parafilm was wrapped around the vial to create an airtight seal, slowing down the rate of particle aggregation. Volumes were measured using Eppendorf Research Plus micro-pipettes with tips replaced after each quantity of solution was extracted. Silica nanoparticles of volume 100 μL were pipetted into 1.5 ml microcentrifuge tubes and placed in a James Products Europe Sonic 3MX Professional Ultrasonic Cleaner sonicator at 37 kHz for 30 minutes at the lowest temperature setting of 17 °C to further reduce the aggregation of silica nanoparticles. Otherwise, there could be observed rotational effects in the spectrum which are undesired. After sonication, the other solution components were added in the following order: water, DNA (if used) and $ZnCl_2$ (if used). The tubes were inverted ten times after each solution was added to ensure thorough mixing. To keep the 25A and 25T oligonucleotides intact, only the silica nanoparticles were placed in the sonicator.

A stepwise approach was taken to account for increasing the complexity of functionalizing the surface of silica nanoparticles. The first step was to trap silica nanoparticles without any surface modifications. This acted as a control and was carried out to ensure proper functionality and alignment of the optical setup. In the second phase, a 100 mM $ZnCl_2$ stock solution was prepared by dissolving 1.363 g of Sigma Aldrich $\geq$ 98% reagent grade $ZnCl_2$ in 100 mL deionized water, then $ZnCl_2$ was added to the silica nanoparticles at a final concentration of 1000 μM. The third stage involved addition of 25A (a 25-mer of deoxyadenosine monophosphate) or 25T (a 25-mer of deoxythymidine monophosphate) to the silica nanoparticle solution with $ZnCl_2$ and then optically trapped. These were selected due to the ability to functionalize silica nanoparticles with fluorescently tagged 25As [1]. 25T was also chosen as it is vastly different in structure to 25A and also proved successful in functionalizing to silica nanoparticles [1]. Both oligonucleotides were ordered from Integrated DNA Technologies (IDT) and resuspended in deionized water to make a 10 μM stock solution. The molecular weight of 25A is 7,768.3 g mol^{-1} and was measured by IDT as having an optical density at the 260 nm wavelength (OD_{260}) of 169.7 which is equal to 559.3 nmol. 559 μL of deiozined water was used to resuspend the 25A to make a 10 μM stock solution. 25T has a molecular weight of 7,542.9 g mol^{-1}. IDT measured the OD_{260} as 117.5 equal to 578.3 nmol. Here, 578 μL of deionized water was pipetted to resuspended the 25T to create the 10 μM stock solution. As the concentration of oligonucleotides at 400 nM and $ZnCl_2$ at 1000 μM yields the maximum loading capacity onto the silica nanoparticles [1], this research used a 400 nM concentration of oligonucleotides in all samples. This relatively low concentration reduced the likelihood of the nebulizer mesh piece becoming clogged. A final step involved combining 25T silica nanoparticles with the following additional concentrations of $ZnCl_2$: 100, 500 and 750 μM. At all stages in the experiment, the oligonucleotides were refrigerated to reduce the rate of degradation.

An Omron MicroAIR U100 Portable Nebulizer (Supplementary Figure 1) was used to

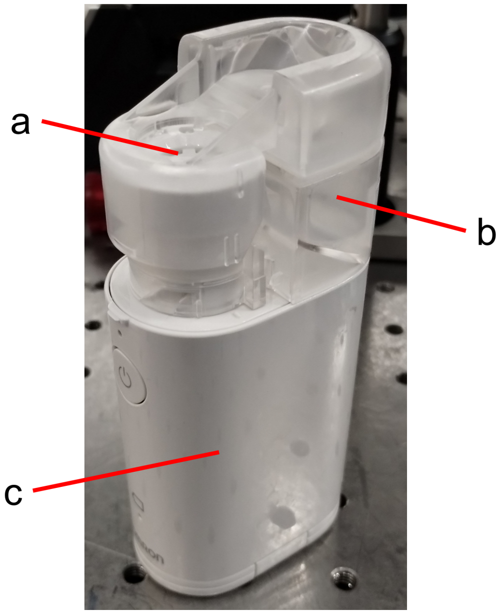

Supplementary Figure 1: **The Omron MicroAIR U100 nebulizer.** There are three main parts: (a) is the interchangeable mesh piece where the aerosol droplets are released, (b) is the reservoir where nanoparticle solutions are placed, and (c) is the battery power source compartment.

release particles into the optical trap. Following each experiment, the reservoir and head mesh pieces were rinsed using deionized water to prevent silica nanoparticles clogging up the mesh such that a sufficient stream of aerosol would be released. Between changing over solution types, the mesh piece was replaced to avoid contamination, particularly of importance when considering the 25A and 25T functionalized particle stages. A particulate respirator was worn at all stages when the nebulizer was in use to avoid inhalation of silica nanoparticles.

1.2 Optical trap laser setup

Previous optical trap experiments [2] used a setup similar to the one in this work. The iris was included such that when the aperture is reduced, the high-power Poisson spot is removed (Supplementary Figure 2), decreasing the signal-to-noise ratio which increases the position resolution of the trapped particle that can be detected by the photodiode. This is necessary as the bright spot in the center of the beam overlaps the scattered field (E_{scat}) and diverging field (E_{div}). The bright Poisson spot forms from the flat mirror edges around the parabolic mirror and was used to confirm the laser alignment. The images in Supplementary Figure 2 were captured by a Point Gray Research CMLN-13S2M-CS infrared camera.

The optical setup is shown in Supplementary Figure 3. Light comes out of a 40 mW NKT Photonics Koheras Basik Mikro 1550 nm laser and is seeded into a Nuphoton High Power Erbium-Doped Fibre Amplifier (EDFA). This is then released from a Thorlabs 3.0 mm diameter collimator and reflects off a mirror, then travels through a Thorlabs $\lambda/2$ @ 1550 nm MULTI-ORDER waveplate set at an angle of 38° to control the power of the trapping

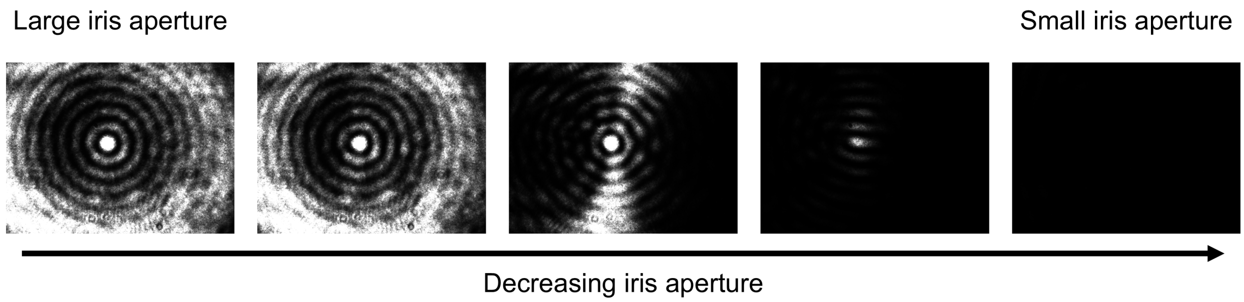

Supplementary Figure 2: **IR images of decreasing iris apertures:** A series of images demonstrating the removal of the Poisson spot with decreasing iris aperture. Closing the iris aperture decreases the size of the Poisson spot until the aperture size is equal to the diameter of the parabolic mirror.

laser beam. The polarizing beam splitter (PBS) trapping beam then enters the Thorlabs $\lambda/4$ @ 1550 nm MULTI-ORDER waveplate set at an angle of 58° to control the polarization of the light and the direction of the diverging and scattered light (E_{div} + E_{scat}). The trapping beam then reflects off an alignment mirror and passes through an iris. Upon the trapping beam entering the vacuum chamber, the light is reflected off a parabolic mirror. This is necessary to tightly focus the beam onto the trapped particle to achieve a higher laser intensity gradient. The E_{div} + E_{scat} beam then travels back through the iris, alignment mirror and $\lambda/4$ waveplate and is sent at right angles to the trapping beam as it goes through the PBS. Following reflection from the final alignment mirror, the E_{div} + E_{scat} beam enters a Thorlabs NDC-50C-4M variable ND filter. This prevents saturation of the Thorlabs 800 – 1700 nm DC – 5 MHz photodetector. The signal is finally displayed on the Rohde and Schwarz RTO2014 oscilloscope.

The laser's power was measured between the alignment mirror and iris using a ThorLabs S146C Photodiode Power Sensor coupled to a ThorLabs PM100D Handheld Optical Power and Energy Meter Console. The power was measured over 100 samples with a mean value of 395.4 mW, a standard deviation of 2.537 mW, a minimum value of 391.3 mW and a maximum value of 400.7 mW.

After particles were released into the vacuum chamber, the pressure was reduced using a vacuum pump. Pressure was measured using an Agilent Technologies FRG-720 pressure gauge. For each experiment, the raw oscilloscope data was aimed to be saved at 3.5 mbar as the PSD produced clean peaks on the oscilloscope for the Lorentzian curve fitting at this pressure. However, due to the manual operation of the controls, it was not possible for the pressure to be exactly 3.5 mbar each time. However, as the pressure gauge has an accuracy of ± 15%, the pressures at which data was saved was within the range of error.

1.3 Data collection and analysis

Data are freely available from the University of Southampton Institutional Repository [3]. Following the collection of raw data from the oscilloscope, the frequency plot of the time domain signal was obtained using a Fourier transform. This was carried out in Python code

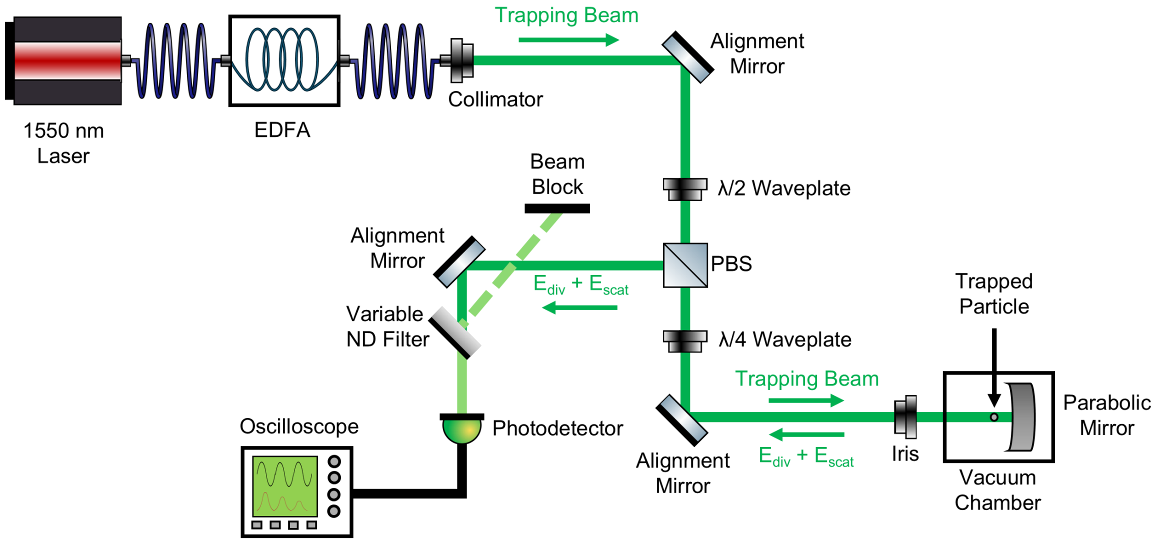

Supplementary Figure 3: **Optical setup:** A diagrammatic representation of the optical setup with the laser system and optical beam path, focusing parabola in vacuum chamber and data collection by an oscilloscope.

using the Optoanalysis package [4]. A Graphical User Interface (GUI) was developed on top of this code to enable seamless data entry as seen in Supplementary Figure 4a. Following the display of the PSD, the recorded pressure was entered, as was the estimated peak frequency of the z, x and y degrees of motion, as well as a Yes/No value if the PSD Lorentzian curve was a good fit (Supplementary Figures 4b-d). The data for each trapped particle was recorded as a row in a comma-separated value (CSV) table.

Silica nanoparticles analyzed

Supplementary Table 1. Values for the particle type and number analyzed in the data analysis section.

Particle Type	Number Analyzed
Standard silica nanoparticle	32
25A 1000 μM $ZnCl_2$ silica nanoparticle	16
25T 1000 μM $ZnCl_2$ silica nanoparticle	16
25T 100 μM $ZnCl_2$ silica nanoparticle	17
25T 500 μM $ZnCl_2$ silica nanoparticle	18
25T 750 μM $ZnCl_2$ silica nanoparticle	15

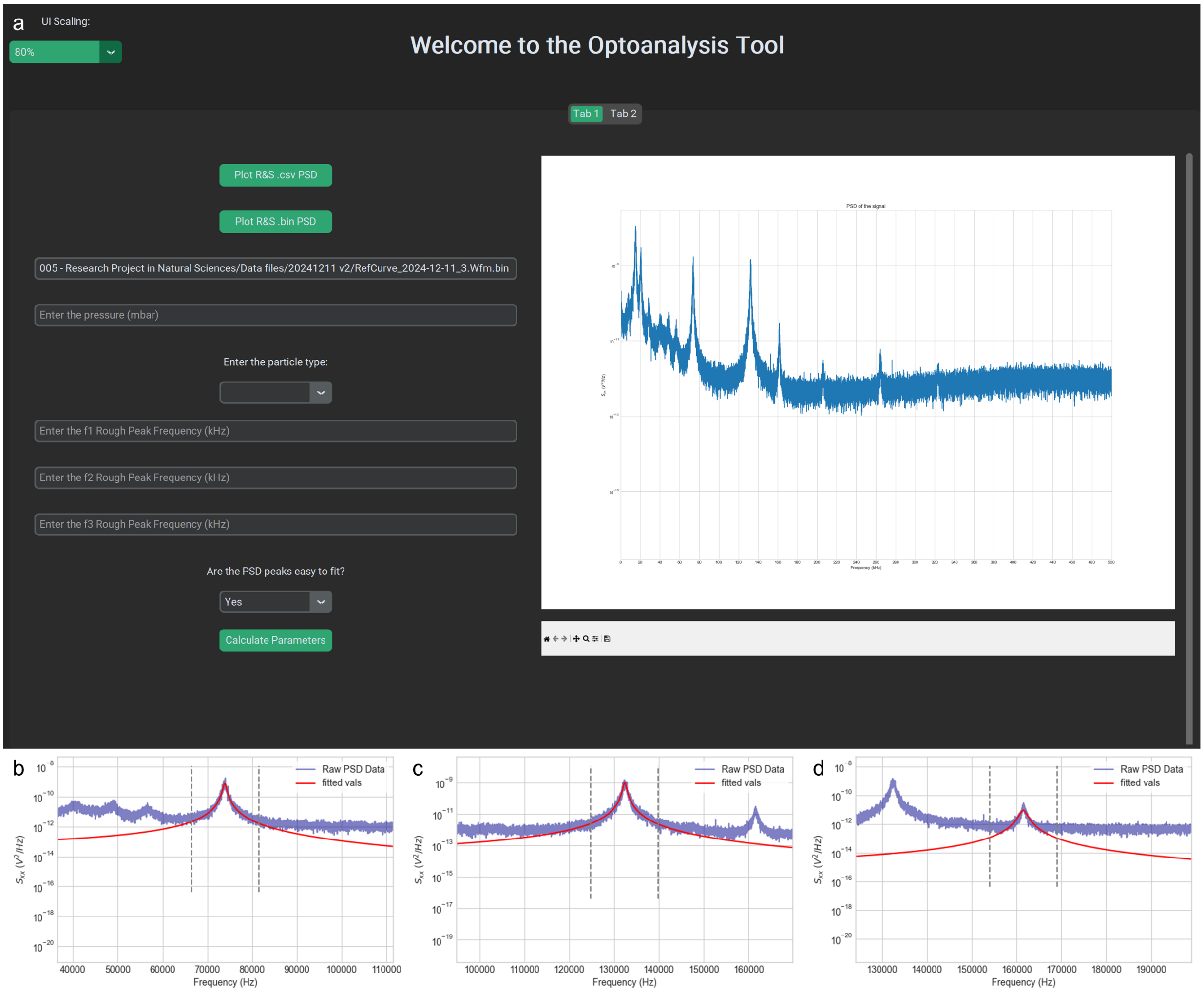

Supplementary Figure 4: **GUI of Optoanalysis** with an example PSD and Lorentzian curve fitting. (a) Screenshot of the Optoanalysis Tool GUI Developed to Enable Data Entry into a CSV Table, (b) Lorentzian curve fitting to the f_1 peak, (c) Lorentzian curve fitting to the f_2 peak and (d) Lorentzian curve fitting to the f_3 peak.

1.4 Process of TEM imaging

The TEM imaging samples were prepared using the same method as for the optical trapping previously described. 5 μL of each sample was pipetted onto a copper grid and left to set for three minutes. Then, the excess solution was wicked away with filter paper. The copper grid with the sample was loaded into a holding rod and inserted into the TEM for imaging. All images were taken using a Hitachi HT7800 TEM.

2 Estimates of the Frequency Shift for Oligonucleotide Adsorption onto Silica in Levitated Optomechanics

2.1 Trap frequencies in optical dipole trap of polarizable particle

This part derives a physics reason for the observed trap frequency shift for the functionalized silica nanoparticles.

2.1.1 Trap frequency formula for z-direction

For a diffraction-limited focused Gaussian laser beam which is forming an optical trap, the trap frequency along the (z) trapping direction is given according to the ponderomotive light-matter interaction by:

$$\omega_{\text{trap}} = \sqrt{\left(\frac{\alpha}{m}\right)\frac{4P}{\pi\epsilon_0 c w_0^4}}, \tag{1}$$

with P the incident trapping laser power, w_0 the beam waist radius at the focal point, m is the mass of the nanoparticle, α its static polarizability, ϵ_0 dielectric constant of vacuum, and c is the speed of light.

Experimental parameters for the optical trap

Laser power $P = 100$ mW $= 0.1$ W, Beam waist $w_0 = 1$ μm $= 1 \times 10^{-6}$m. For adenine and thymine mass and static polarizability, see Table 2. This leads to a z-trap frequency $f_0 = 135\,\text{kHz}$.

Supplementary Table 2. Literature values for mass and polarizability for two nucleobases from DFT and Raman experiments.

Nucleobase	Polarizability α (C·m^2/V)	Mass m (kg)	$\frac{\alpha}{m}$ (C·m^2/V·kg)
Adenine	1.65×10^{-39}	2.24×10^{-25}	7.73×10^{-15}
Thymine	1.48×10^{-39}	2.09×10^{-25}	7.08×10^{-15}

2.1.2 Computation and experiments for dielectric properties of oligonucleotides

Polarizability type: Static (frequency-independent) polarizabilities are computed as response properties or finite field calculations. There have been extensive ab-initio density functional theory (DFT) calculations using various sets of density functionals [5, 6, 7] and complemented by Hartee-Fock methods [8], and classical electrodynamical calculations by Kramers-Kronig relations [9, 10].

Experiments on dielectric properties of oligonucleotides by Rayleigh scattering [11], by electrostatic force microscopy [12], and by optical extinction coefficient measurements [13].

Silica nanoparticle parameters

Silica particle diameter $d = 100$ nm results in particle mass of $m_{silica} = 1.15 \times 10^{-18}$ kg and a static polarizabilty is estimated using Clausius-Mossotti relation for a silica refractive index at 1550 nm of $n \approx 1.44$. This results in: $\alpha_{silica} = 3.66 \times 10^{-33}$ C · m^2/V .

2.2 Trap frequency change for oligonucleotide coated silica nanoparticles

One monolayer of adenine gives about 1 kHz of frequency shift (0.74% shift in trap frequency with respect to the uncoated silica case) in the optical trap of a 100 nm diameter silica nanoparticle. The increase in monolayers (ML) is linear in the frequency shift in the regime of small perturbations. These are small effect, but resolvable in levitated optomechanics optical traps.

Parameters for 1 monolayer (1ML) of adenine on a silica nanoparticle

Number of adenine molecules in monolayer coating a spherical 100 nm diameter silica nanoparticle is $N = 3.14 \times 10^4$. The total polarizability of 1ML of adenine is: $\alpha_{A,total} = N \times \alpha_A = 5.18 \times 10^{-35}\, C \cdot m^2/V$. The mass of one adenine monolayer: $m_A = N \times 2.24 \times 10^{-25}$ kg $= 7.03 \times 10^{-21}$ kg. Total polarizability of an adenine coated nanoparticle is:

$$\alpha_{total} = \alpha_{silica} + \alpha_{A,total} = 3.66 \times 10^{-33} + 5.18 \times 10^{-35} \approx 3.71 \times 10^{-33}.$$

We derive a trap frequency ratio of $\frac{\omega_{total}}{\omega_{silica}} = 1.007$. By analogous calculation, we derive a smaller frequency shift for thymine for 1ML, namely $\omega_T = 0.93\omega_A$.

2.3 Frequency shift for a single adenine/thymine molecule

The frequency shift for small perturbation is derived by the trap frequency ratio with the molecule adsorbed:

$$\frac{\omega}{\omega_0} = \sqrt{\frac{\alpha_{silica} + \alpha_{molecule}}{m_{silica} + m_{molecule}} \left(\frac{\alpha_{silica}}{m_{silica}}\right)^{-1}}.$$

Since $m_{molecule} \ll m_{silica}$, we approximate:

$$\frac{\omega}{\omega_0} \approx \sqrt{1 + \frac{\alpha_{molecule}}{\alpha_{silica}}},$$

and the relative frequency change:

$$\frac{\Delta\omega}{\omega_0} \approx \frac{1}{2}\frac{\alpha_{molecule}}{\alpha_{silica}}.$$

This results in relative frequency shifts for a single adenine molecule of: $\frac{\Delta\omega}{\omega_0} \approx 2.25 \times 10^{-7}$, which corresponds to an absolute frequency shift of, $\Delta f_A = f_0 \frac{\Delta\omega}{\omega_0} = 0.030$ Hz, and for thymine to $\Delta f_T = 0.028$ Hz.

Supplementary References

[1] Yue Huang, Jiehua Ma, Yuting Yan, and Chao Li. Metal-bridged dna-functionalized silica nanoparticles for multifacet biological applications. *Microchemical Journal*, 173:107017, 2022.

[2] Jamie Alexander Vovrosh. *Parametric feedback cooling and squeezing of optically levitated particles*. PhD thesis, University of Southampton, 2018.

[3] Timothy Wilson and Hendrik Ulbricht. Raw oscilloscope data for the paper 'oligonucleotide selective detection by levitated optomechanics', July 2025.

[4] Markus Rademacher and Ashley Setter. Ashleysetter/optoanalysis: v4. 0.1 release, 2017.

[5] Jürgen Hafner. Ab-initio simulations of materials using vasp: Density-functional theory and beyond. *Journal of computational chemistry*, 29(13):2044–2078, 2008.

[6] Yang Yang, Ka Un Lao, David M Wilkins, Andrea Grisafi, Michele Ceriotti, and Robert A DiStasio Jr. Quantum mechanical static dipole polarizabilities in the qm7b and alphaml showcase databases. *Scientific data*, 6(1):152, 2019.

[7] Dongbo Zhao, Shubin Liu, and Dahua Chen. A density functional theory and information-theoretic approach study of interaction energy and polarizability for base pairs and peptides. *Pharmaceuticals*, 15(8):938, 2022.

[8] S Gowtham, Ralph H Scheicher, Rajeev Ahuja, Ravindra Pandey, and Shashi P Karna. Physisorption of nucleobases on graphene: Density-functional calculations. *Physical Review B—Condensed Matter and Materials Physics*, 76(3):033401, 2007.

[9] Anatoliy Pinchuk. Optical constants and dielectric function of dna's nucleotides in uv range. *Journal of Quantitative Spectroscopy and Radiative Transfer*, 85(2):211–215, 2004.

[10] Denis Fichou. *Handbook of oligo-and polythiophenes*. John Wiley & Sons, Hoboken, NJ, 2008.

[11] Christian Jonin, Estelle Salmon, and Pierre-François Brevet. Hyper-rayleigh scattering of adenine, thymine, and cytosine in neat water. *The Journal of Chemical Physics*, 155(20), 2021.

[12] Ana Cuervo, Pablo D Dans, José L Carrascosa, Modesto Orozco, Gabriel Gomila, and Laura Fumagalli. Direct measurement of the dielectric polarization properties of dna. *Proceedings of the National Academy of Sciences*, 111(35):E3624–E3630, 2014.

[13] Seyedeh Maryam Banihashemian, Vengadesh Periasamy, Seyed Mohammad Hossein Mousa Kazemi Mohammadi, Richard Ritikos, and Saadah Abdul Rahman. Optical characterization of oligonucleotide dna influenced by magnetic fields. *Molecules*, 18(10):11797–11808, 2013.
