# Supplementary figures and images for: Oligonucleotide Selective Detection by Levitated Optomechanics
Source: ACS Nanosci Au. 2025 Oct 20;6(1):28–34. doi: 10.1021/acsnanoscienceau.5c00128 (PMC12921583; doi:10.1021/acsnanoscienceau.5c00128)

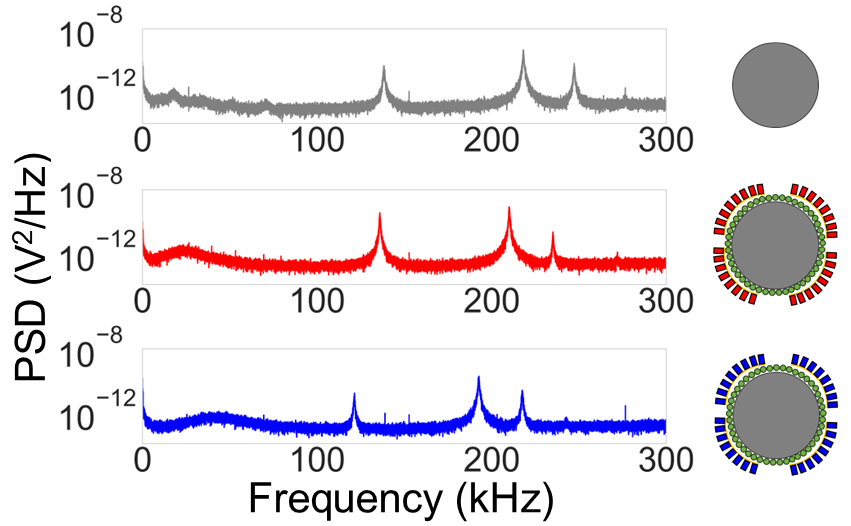

Supplement: Supplementary file 2 [file ng5c00128_si_002.zip › SupportInfo/TOC_updated.png]

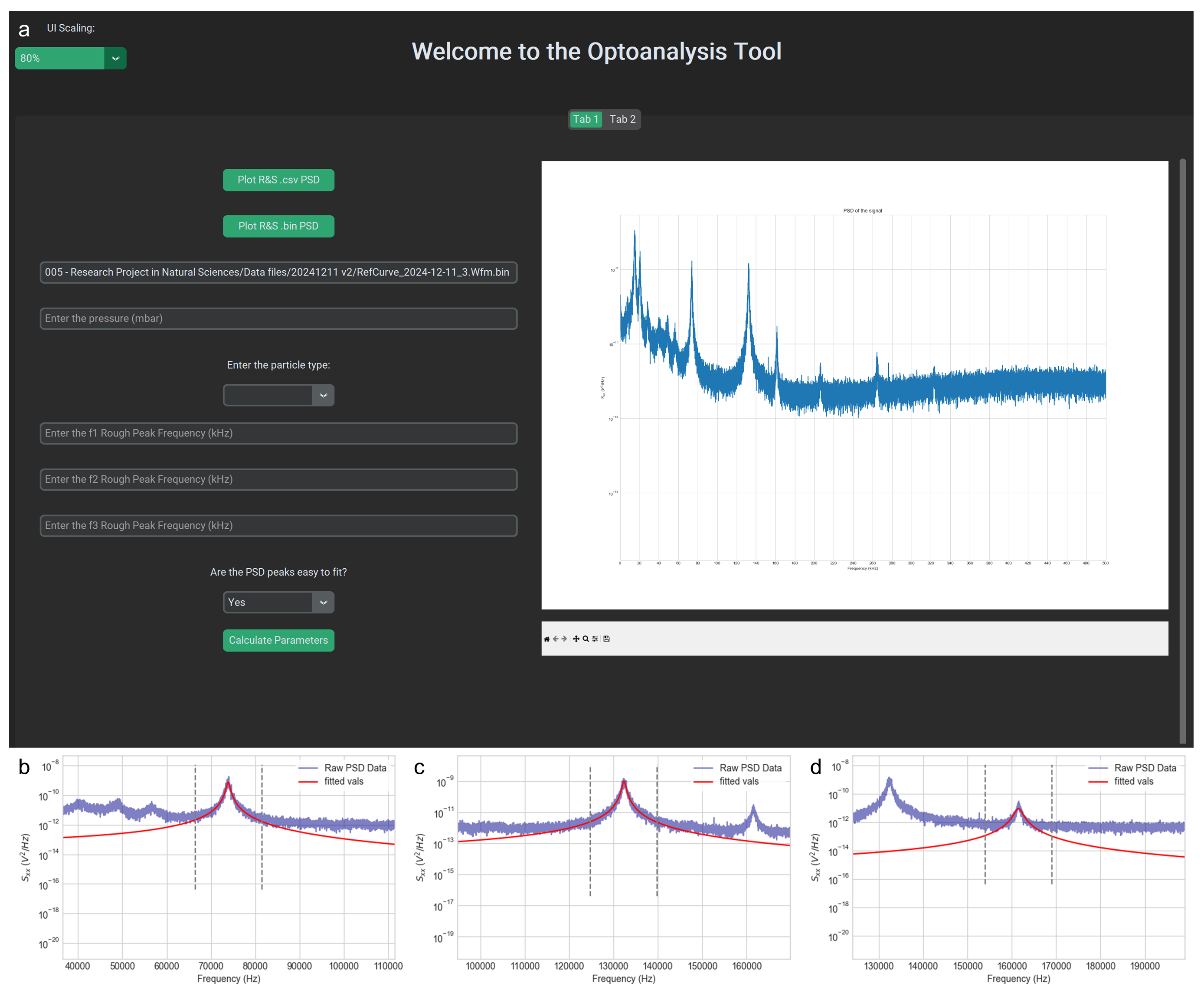

Supplement: Supplementary file 2 [file ng5c00128_si_002.zip › SupportInfo/optotool_updated.png]

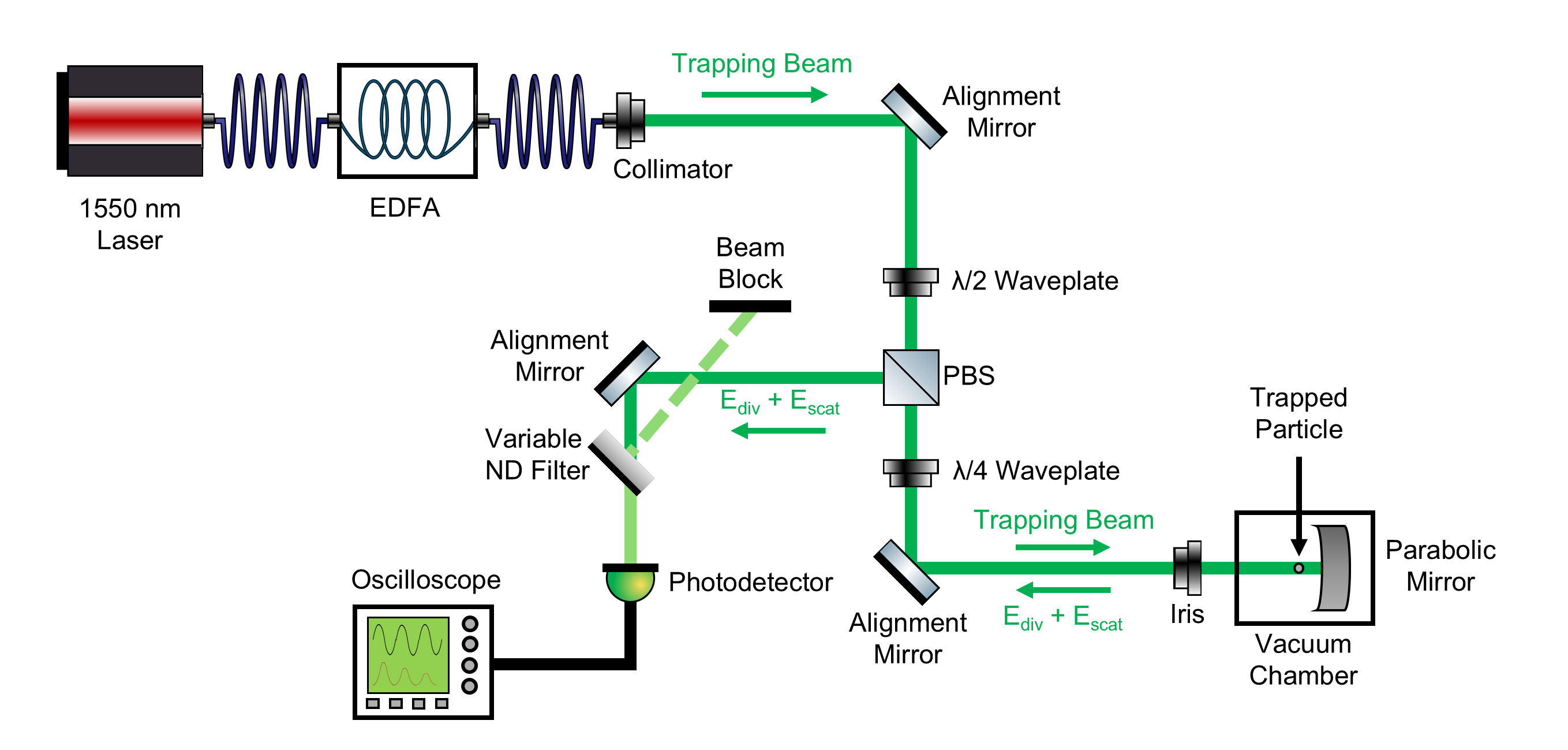

Supplement: Supplementary file 2 [file ng5c00128_si_002.zip › SupportInfo/setup.png]

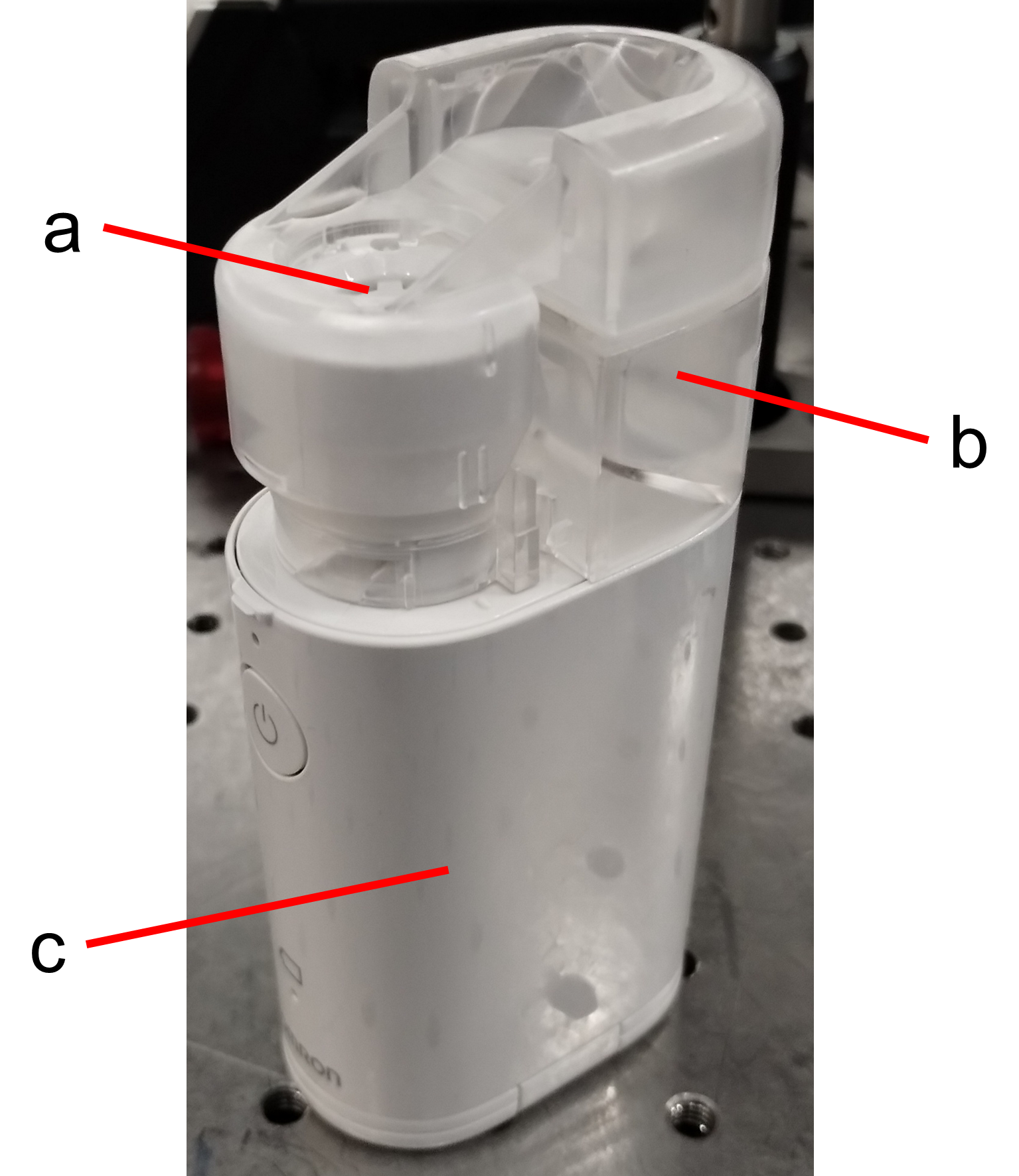

Supplement: Supplementary file 2 [file ng5c00128_si_002.zip › SupportInfo/nebulizer.png]
